# Association between intake of flavanones and the overweight/obesity and central obesity in children and adolescents: a cross-sectional study from the NHANES database

**DOI:** 10.3389/fnut.2024.1430140

**Published:** 2024-07-17

**Authors:** Yangyang Liu, Zhuoqiong Liu, Nan Wu

**Affiliations:** ^1^Developmental Behavior Pediatrics, The Sixth Affiliated Hospital of Harbin Medical University, Harbin, China; ^2^Child Health Section, The Sixth Affiliated Hospital of Harbin Medical University, Harbin, China

**Keywords:** flavanones, children and adolescence, overweight & obesity, central obesity, NHANES

## Abstract

**Aim:**

The prevalence of obesity (Ob), overweight (Ow) and central obesity (CO) in children and adolescents has increased dramatically over the past decades globally. Flavanones have been recently studied as adjuvants for the treatment of obesity. This study was aimed at evaluating the association between intake of flavanones and its subclasses and the Ow/Ob and CO in children and adolescents.

**Methods:**

This cross-sectional study extracted the data of children and adolescents with Ow/Ob and CO from the National Health and Nutrition Examination Survey (NHANES) database for 2007–2010 and 2017–2018. Ow and Ob were defined as a body mass index (BMI) ≥ 85^th^ percentile. CO was defined as a waist circumference (WC) ≥ 90th percentile. The association between intake of flavanones and its subclasses and the Ow/Ob and CO in children and adolescents was determined by weighted univariate and multivariate Logistic regression models adjusted for potential covariates, and odds ratios (ORs) with 95% confidence intervals (CIs) was calculated. To further explore association between intake of flavanones and its subclasses and the Ow/Ob and CO in children and adolescents, subgroup analyses stratified by age, and gender.

**Results:**

Of the total 5,970 children and adolescents, 2,463 (41.2%) developed Ow/Ob and 1,294 (21.7%) patients developed CO. High intake of flavanones, eriodictyol, hesperetin, and naringenin were associated with lower odds of Ow/Ob in children and adolescents. (OR: 0.75, 95%CI: 0.62–0.92, OR: 0.69, 95%CI: 0.55–0.87, OR: 0.69, 95%CI: 0.55–0.87, and OR: 0.76, 95%CI: 0.63–0.92, respectively). In addition, high intake of flavanones, eriodictyol, and naringenin were associated with lower odds of CO in children and adolescents (OR: 0.71, 95%CI: 0.57–0.88, OR: 0.67, 95%CI: 0.51–0.86, and OR: 0.69, 95%CI: 0.55–0.86, respectively). Subgroup analyses showed that among all the different subgroups, high intake of flavanones was associated with lower odds of Ow/Ob and CO in children and adolescents.

**Conclusion:**

A diet loaded with high flavanones were associated with lower odds of Ow/Ob and CO in children and adolescents, and children and adolescents should be encouraged to increase their intake of flavanones.

## Introduction

Childhood obesity (Ob) is one of the most serious worldwide public health problems in the twenty-first century (1). According to the data of WHO in 2022, 37 million children were overweight of those aged <5 years, and 390 million of aged 5–19 years (2). Overweight (Ow) and Ob are indirect indicators based on body mass index (BMI, kg/m^2^), indicating excessive accumulations of fat, while central obesity (CO) is an indirect indicator based on waist circumference, indicating excessive accumulation of visceral fat around the stomach and abdomen (3). Ow, Ob and CO have been reported to be associated with adverse outcomes in many diseases, such as metabolic syndrome, type 2 diabetes, and cardiovascular disease (3–5). Being overweight in children and adolescents affects their immediate health, has adverse psychosocial consequences, and is associated with greater risk and earlier onset of various non-communicable diseases, it is crucial to adopt preventive interventions. Accumulating evidence indicates that low-quality diets were associated with an increased risk of Ow, Ob, and CO (4, 6, 7). It is essential to recommend dietary modification as the primary approach for the prevention and intervention of childhood Ob.

Flavanone, a common subclass of flavonoids, is a natural phenolic compound with diverse biological effects, mostly found in citrus fruits, mainly including hesperidin, naringenin, eriodictyol, etc. (8, 9). Mounting evidence has demonstrated that flavanones exert multiple therapeutic effects including anti-inflammation (10, 11), antioxidant (12, 13), anti-cancer (14, 15), cardioprotection (16, 17), anti-diabetic (18), and anti-obesity (19, 20). Vernarelli et al. (21) found that in U.S. adults, intake of flavonoids, including flavanones, was inversely associated with Ob and C-reactive protein levels (a marker of inflammation) in women. Studies in Korean adults have found that a high intake of flavanones may be associated with a decreased body fat percentage and CO in women (22). In addition, several animal experimental studies have found that hesperidin, naringenin, and eriodictyol can improve lipid metabolism and control body weight and Ob (10, 18, 19, 23, 24). To our knowledge, however, most of the current studies have focused on adults and animals, and no studies on the association of flavanones and its subclasses, with Ow/Ob and CO in children and adolescents.

This study is based on the NHANES database to analyze the association of flavanones and its subclasses intake, with Ow/Ob and CO in children and adolescents, and whether this association remains in patients stratified by age, and gender.

## Methods

### Study design and population

This cross-sectional study extracted the data of children and adolescents with Ow/Ob and CO from the National Health and Nutrition Examination Survey (NHANES) database for 2007–2010 and 2017–2018. The NHANES is a nationally representative survey conducted by the National Center for Health Statistics (NCHS) of the Centers for Disease Control and Prevention (CDC), that surveys health, nutrition, and other lifestyle factors across the adults and children in the United States (25). Complete details about the NHANES data collection and interview process are available on the NHANES website.^1^ NHANES is a publicly available dataset and was approved by the NCHS Ethics Review Board, and all patients/participants provided their written informed consent.

The inclusion criteria were: (1) patients aged 6–18 years old. The exclusion criteria were: (1) patients with missing data on energy and flavanones intake; (2) patients with missing data on height, weight, and waist circumference.

### Covariates

Covariates included age (in years), gender (female/male), race (Non-Hispanic White, Non-Hispanic Black, or others), educational level (<9th grade, or ≥ 9th grade), poverty income ratio (PIR) (<1.0, ≥1.0, or unknown), household education level (less than high school degree, or high school degree and above), mother smoked when pregnant, total energy, fiber, total fat, birth weight (<5.5, 5.5–8.9, ≥9 or unknown), tobacco exposure were defined as subjects who had responded “Yes” to the question “Does anyone smoke in the home/of people who live here smoke tobacco/of people who smoke inside this home? Physical activity level was classified as under 12 years old (days physically active at least 60 min), over 12 years old high level (any one of the vigorous work activity and vigorous recreational activities), medium level (any two of moderate work activity and walk or bicycle and moderate recreational activities).

### Outcome variables

The recommended body mass index (BMI) percentiles from CDC for children with different age and gender were used.^2^ Ow and Ob were defined as a BMI ≥ 85th percentile (26). Waist circumference (WC) was measured at the thinnest point of the abdomen at the end of a normal expiration. CO was defined as a WC ≥ 90th percentile (27).

### Main explanatory variable

The main explanatory variables were the intake of flavanones and its subclasses.

Total flavanones intake (mg/1000 kcal) = Flavanones/Total energy*1000 (mg/1000 kcal).

Total eriodictyol intake (mg/1000 kcal) = Eriodictyol/Total energy*1000 (mg/1000 kcal).

Total hesperetin intake (mg/1000 kcal) = Hesperetin /Total energy*1000 (mg/1000 kcal).

Total naringenin intake (mg/1000 kcal) = Naringenin/Total energy*1000 (mg/1000 kcal).

Flavanones, eriodictyol, hesperetin, and naringenin levels were assigned using the following ranges based on previous study (28):

Flavanones: grade 1 (level = 0 mg/1000 kcal), grade 2 (0<level ≤ 1.74 mg/1000 kcal), grade 3 (level > 1.74 mg/1000 kcal).
Eriodictyol: grade 1 (level = 0 mg/1000 kcal), grade 2 (0<level ≤ 0.09 mg/1000 kcal), grade 3 (level > 0.09 mg/1000 kcal).
Hesperetin: grade 1 (level = 0 mg/1000 kcal), grade 2 (0<level ≤ 7.17 mg/1000 kcal), grade 3 (level > 7.17 mg/1000 kcal).
Naringenin: grade 1 (level = 0 mg/1000 kcal), grade 2 (0<level ≤ 0.32 mg/1000 kcal), grade 3 (level > 0.32 mg/1000 kcal).

### Statistical analysis

Continuous variables were described as the mean ± standard error [Mean(±SE)], and compared using Student’s *t* test. Categorical variables were represented as number (n) and percentage, and Chi-square test was used for comparison between groups.

Random forests were used to impute missing values. Sensitivity analyses were performed on the data before and after imputation (Supplementary Table S1). Weighted univariate and multivariate Logistic regression models were used for analyzing the association between the intake of flavanones and the Ow/Ob and CO in children and adolescents, and odds ratios (ORs) with 95% confidence intervals (CIs) were calculated. Model I [a] and Model I [b] as well as Model II [a] and Model II [b] were adjusted based on the statistically significant variables in Supplementary Tables S2, S3 by step-based regression, respectively. Model I [a] and Model I [b] finally adjusted for biological factors of age, gender, and race. The Model II [a] and Model II [b] finally adjusted for all confounding factors including age, gender, race, education, PIR, household education, tobacco exposure, birth weight, and mother smoking when pregnant. Subgroup analysis was performed stratified by age (6 < age ≤ 11 years, or 12 < age ≤ 18 years), and gender (female or male).

Data cleaning, missing value imputation, and modeling were performed using Python 3.9 (Python Software Foundation, Delaware, United States). Statistical analysis and sensitivity analyses were performed using SAS 9.4 software (SAS Institute Inc., Cary, NC, United States). The *p*-values <0.05 was considered significant and 0.05<*p*-values <0.1 was considered marginal significance for all analyses.

## Results

### Patients’ characteristics

**Figure 1:**
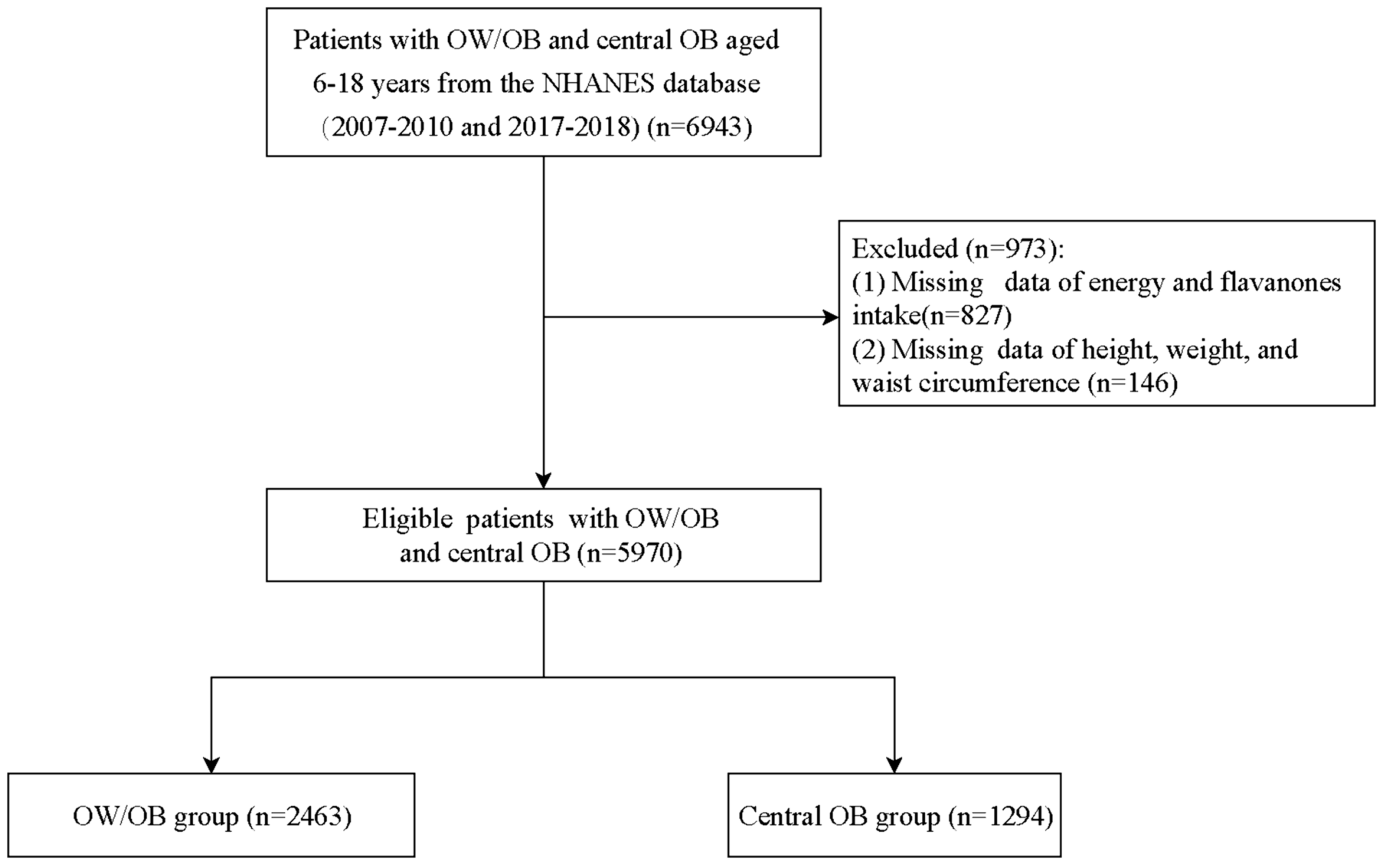
Subject screening flowchart.

According to the inclusion and exclusion criteria in Figure 1, 5,970 eligible children and adolescents were extracted for analysis, of which 2,463 (41.2%) patients developed Ow/Ob and 1,294 (21.7%) patients developed CO. Table 1 compares the baseline characteristics of non-Ow/Ob group and Ow/Ob group, as well as non-CO group and CO group. The proportion of eriodictyol intake of 0 was significantly higher in the Ow/Ob group than in the non-Ow/Ob group (77.98% vs. 73.71%, *p* = 0.037). Ow/Ob group was more likely to be non-Hispanic Black, have less than 9-grade education, PIR<1.0, and household education level less than high school degree. There were significant differences between the Ow/Ob group and non-Ow/Ob group with respect to birth weight, tobacco exposure, and mother smoking when pregnant (*p* < 0.05 for all). The proportion of flavanones intake of 0 and naringenin intake of 0 was significantly higher in the CO group than in the non-CO group (49.45% vs. 43.65%, *p* = 0.029, and 49.83% vs. 44.06%, *p* = 0.023). CO group was younger, more likely to be Non-Hispanic Black, have less than 9 grade education, PIR < 1.0, household education level less than high school degree. There were significant differences between the CO group and non-CO group with respect to birth weight, tobacco exposure, and mother smoking when pregnant (*p* < 0.05 for all).

**Table 1:** Characteristics of children and adolescents with Ow/Ob and Central OB.

Variables	Total (*n* = 5,970)	Ow/Ob		Statistics	*p*	Central OB		Statistics	*p*
		No (*n* = 3,507)	Yes (*n* = 2,463)			No (*n* = 4,676)	Yes (*n* = 1,294)		
Flavanones, mg, Mean (S.E)	12.23 (0.77)	12.61 (0.99)	11.62 (0.82)	*t* = 0.92	0.361	12.68 (0.88)	10.42 (0.89)	t = 2.07	0.044
Flavanones, mg/1000 kcal, Mean (S.E)	6.52 (0.35)	6.48 (0.46)	6.59 (0.49)	*t* = −0.17	0.867	6.72 (0.42)	5.74 (0.45)	t = 1.64	0.108
Flavanones, *n* (%)				χ^2^ = 2.52	0.284			χ^2^ = 7.08	0.029
0 mg/1000 kcal	2,598 (44.81)	1,496 (43.67)	1,102 (46.64)			2000 (43.65)	598 (49.45)		
0–1.74 mg/1000 kcal	1,568 (27.62)	961 (28.00)	607 (27.00)			1,252 (28.12)	316 (25.59)		
>1.74 mg/1000 kcal	1804 (27.58)	1,050 (28.33)	754 (26.36)			1,424 (28.23)	380 (24.96)		
Eriodictyol, mg, Mean (S.E)	0.17 (0.02)	0.18 (0.03)	0.17 (0.03)	*t* = 0.31	0.759	0.18 (0.03)	0.14 (0.02)	t = 1.29	0.203
Eriodictyol, mg/1000 kcal, Mean (S.E)	0.08 (0.01)	0.09 (0.01)	0.08 (0.01)	*t* = 0.21	0.837	0.09 (0.01)	0.07 (0.01)	t = 1.48	0.146
Eriodictyol, *n* (%)				χ^2^ = 6.61	0.037			χ^2^ = 3.86	0.145
0 mg/1000 kcal	4,320 (75.34)	2,523 (73.71)	1797 (77.98)			3,361 (74.67)	959 (78.01)		
0–0.09 mg/1000 kcal	762 (12.33)	465 (13.33)	297 (10.72)			604 (12.52)	158 (11.57)		
>0.09 mg/1000 kcal	888 (12.33)	519 (12.97)	369 (11.30)			711 (12.81)	177 (10.41)		
Hesperetin, mg, Mean (S.E)	9.36 (0.60)	9.70 (0.81)	8.81 (0.60)	*t* = 1.01	0.319	9.70 (0.69)	7.99 (0.66)	t = 2.00	0.051
Hesperetin, mg/1000 kcal, Mean (S.E)	4.93 (0.26)	4.91 (0.35)	4.98 (0.36)	*t* = −0.14	0.887	5.05 (0.31)	4.44 (0.36)	t = 1.31	0.196
Hesperetin, *n* (%)				χ^2^ = 5.78	0.056			χ^2^ = 4.17	0.124
0 mg/1000 kcal	3,709 (65.24)	2,150 (63.85)	1,559 (67.49)			2,876 (64.42)	833 (68.52)		
0–7.17 mg/1000 kcal	1,144 (17.38)	715 (18.56)	429 (15.45)			917 (17.91)	227 (15.22)		
>7.17 mg/1000 kcal	1,117 (17.39)	642 (17.59)	475 (17.06)			883 (17.67)	234 (16.26)		
Naringenin, mg, Mean (S.E)	2.70 (0.20)	2.73 (0.22)	2.64 (0.25)	*t* = 0.36	0.724	2.80 (0.22)	2.30 (0.28)	t = 1.69	0.099
Naringenin, mg/1000 kcal, Mean (S.E)	1.50 (0.11)	1.49 (0.13)	1.53 (0.15)	*t* = −0.23	0.818	1.57 (0.13)	1.23 (0.11)	t = 2.23	0.031
Naringenin, *n* (%)				χ^2^ = 3.37	0.186			χ^2^ = 7.53	0.023
0 mg/1000 kcal	2,617 (45.21)	1,504 (44.15)	1,113 (46.94)			2012 (44.06)	605 (49.83)		
0–0.32 mg/1000 kcal	1,630 (27.27)	993 (27.48)	637 (26.92)			1,303 (27.72)	327 (25.43)		
>0.32 mg/1000 kcal	1723 (27.52)	1,010 (28.38)	713 (26.14)			1,361 (28.22)	362 (24.74)		
Age, years, Mean (S.E)	12.07 (0.07)	12.06 (0.09)	12.09 (0.10)	*t* = −0.23	0.817	12.11 (0.08)	11.90 (0.10)	t = 2.07	0.044
Gender, *n* (%)				χ^2^ = 0.91	0.341			χ^2^ = 0.64	0.425
Male	3,034 (50.08)	1807 (49.32)	1,227 (51.32)			2,425 (50.38)	609 (48.90)		
Female	2,936 (49.92)	1700 (50.68)	1,236 (48.68)			2,251 (49.62)	685 (51.10)		
Race, *n* (%)				χ^2^ = 29.85	<0.001			χ^2^ = 13.07	0.001
Non-Hispanic White	1883 (56.34)	1,200 (59.91)	683 (50.55)			1,513 (57.91)	370 (50.01)		
Non-Hispanic Black	1,389 (13.70)	784 (12.76)	605 (15.22)			1,090 (13.43)	299 (14.78)		
Others	2,698 (29.96)	1,523 (27.33)	1,175 (34.23)			2073 (28.66)	625 (35.21)		
Education, *n* (%)				χ^2^ = 7.68	0.006			χ^2^ = 30.47	<0.001
<9th grade	4,842 (78.49)	2,831 (77.07)	2011 (80.77)			3,735 (76.97)	1,107 (84.59)		
≥9th grade	1,128 (21.51)	676 (22.93)	452 (19.23)			941 (23.03)	187 (15.41)		
PIR, *n* (%)				χ^2^ = 22.80	<0.001			χ^2^ = 13.74	0.001
<1.0	1,680 (21.42)	936 (18.76)	744 (25.73)			1,294 (19.87)	386 (27.65)		
≥1.0	3,791 (70.50)	2,298 (73.93)	1,493 (64.94)			3,003 (72.22)	788 (63.57)		
Unknown	499 (8.09)	273 (7.32)	226 (9.33)			379 (7.91)	120 (8.78)		
Household education, *n* (%)				χ^2^ = 19.78	<0.001			χ^2^ = 10.86	<0.001
<high school degree	1,639 (19.29)	870 (16.39)	769 (24.00)			1,224 (17.99)	415 (24.55)		
≥high school degree	4,331 (80.71)	2,637 (83.61)	1,694 (76.00)			3,452 (82.01)	879 (75.45)		
Tobacco exposure, *n* (%)				χ^2^ = 6.12	0.013			χ^2^ = 14.10	<0.001
No	5,134 (87.14)	3,028 (88.41)	2,106 (85.08)			4,036 (88.45)	1,098 (81.87)		
Yes	836 (12.86)	479 (11.59)	357 (14.92)			640 (11.55)	196 (18.13)		
Physical activity, *n* (%)				χ^2^ = 0.91	0.635			χ^2^ = 3.76	0.152
No	551 (8.65)	315 (8.60)	236 (8.74)			424 (8.31)	127 (10.01)		
Yes	3,469 (59.51)	2058 (60.11)	1,411 (58.53)			2,756 (60.25)	713 (56.51)		
Unknown	1950 (31.84)	1,134 (31.29)	816 (32.73)			1,496 (31.43)	454 (33.48)		
Birth weight, *n* (%)				χ^2^ = 11.64	0.009			χ^2^ = 26.59	<0.001
<5.5 pounds	584 (9.40)	372 (9.64)	212 (9.00)			460 (9.04)	124 (10.82)		
5.5 ~ 8.9 pounds	3,592 (56.84)	2086 (55.75)	1,506 (58.62)			2,764 (56.22)	828 (59.35)		
≥9 pounds	381 (7.23)	185 (6.37)	196 (8.63)			262 (6.45)	119 (10.39)		
Unknown	1,413 (26.53)	864 (28.24)	549 (23.74)			1,190 (28.29)	223 (19.44)		
Mother smoked when pregnant *n* (%)				χ^2^ = 5.89	0.015			χ^2^ = 24.16	<0.001
No	5,324 (88.36)	3,160 (89.67)	2,164 (86.24)			4,211 (89.50)	1,113 (83.79)		
Yes	646 (11.64)	347 (10.33)	299 (13.76)			465 (10.50)	181 (16.21)		
Total energy, kcal, Mean (S.E)	2009.07 (16.84)	2027.71 (18.63)	1978.88 (28.61)	*t* = 1.52	0.136	2021.05 (17.12)	1960.90 (40.68)	t = 1.43	0.160
Fiber, gm, Mean (S.E)	14.04 (0.20)	14.22 (0.23)	13.75 (0.30)	*t* = 1.41	0.165	14.07 (0.22)	13.90 (0.40)	t = 0.39	0.701
Total fat, gm, Mean (S.E)	75.93 (0.82)	76.17 (0.82)	75.54 (1.43)	*t* = 0.42	0.675	76.21 (0.79)	74.80 (1.84)	t = 0.79	0.434

t, t-test; χ^2^, Chi-square test; −, Fisher exact; S.E, standard error; Ow, overweight; Ob, obesity; Central OB, central obesity; PIR, poverty income ratio.

### Associations of flavanones intake with ow/Ob and CO in children and adolescents

Compared with no intake of flavanones and its subclasses, intake of flavanones (level > 1.74), eriodictyol (0<level ≤ 0.09 and level > 0.09), hesperetin (0 < level ≤ 7.17), and naringenin (level > 0.32), were associated with lower odds of Ow/Ob in children and adolescents (OR: 0.75, 95%CI: 0.62–0.92, OR: 0.70, 95%CI: 0.52–0.94, OR: 0.69, 95%CI: 0.55–0.87, OR: 0.69, 95%CI: 0.55–0.87, and OR: 0.76, 95%CI: 0.63–0.92, respectively) (Table 2). In addition, compared with no intake of flavanones and its subclasses, intake of flavanones (level > 1.74), eriodictyol (level > 0.09), hesperetin (0 < level ≤ 7.17), and naringenin (level > 0.32) were associated with lower odds of CO in children and adolescents (OR: 0.71, 95%CI: 0.57–0.88, OR: 0.67, 95%CI: 0.51–0.86, OR: 0.73, 95%CI: 0.54–0.98, and OR: 0.69, 95%CI: 0.55–0.86, respectively) (Table 2).

**Table 2:** Associations of flavanones and its subclasses intake with Ow/Ob and Central OB in children and adolescents.

Variables	Ow/Ob				Central OB			
	Model I^[a]^		Model II^[b]^		Model I^[a]^		Model II^[b]^	
	OR (95%CI)	*P*	OR (95%CI)	*P*	OR (95%CI)	*P*	OR (95%CI)	*P*
Flavanones, *n* (%)								
0 mg/1000 kcal	Ref		Ref		Ref		Ref	
0–1.74 mg/1000 kcal	0.90 (0.74–1.10)	0.295	0.85 (0.69–1.05)	0.120	0.79 (0.62–0.99)	0.049	0.75 (0.59–0.95)	0.020
>1.74 mg/1000 kcal	0.82 (0.70–0.96)	0.016	0.75 (0.62–0.92)	0.007	0.73 (0.59–0.89)	0.003	0.71 (0.57–0.88)	0.002
Eriodictyol, *n* (%)								
0 mg/1000 kcal	Ref		Ref		Ref		Ref	
0–0.09 mg/1000 kcal	0.73 (0.57–0.94)	0.017	0.70 (0.52–0.94)	0.020	0.84 (0.65–1.10)	0.196	0.86 (0.65–1.14)	0.289
>0.09 mg/1000 kcal	0.78 (0.62–0.98)	0.031	0.69 (0.55–0.87)	0.002	0.74 (0.56–0.98)	0.036	0.67 (0.51–0.86)	0.003
Hesperetin, *n* (%)								
0 mg/1000 kcal	Ref		Ref		Ref		Ref	
0–7.17 mg/1000 kcal	0.77 (0.62–0.95)	0.018	0.69 (0.55–0.87)	0.003	0.77 (0.58–1.02)	0.067	0.73 (0.54–0.98)	0.037
>7.17 mg/1000 kcal	0.85 (0.72–1.01)	0.066	0.83 (0.66–1.03)	0.085	0.80 (0.66–0.98)	0.035	0.82 (0.65–1.03)	0.081
Naringenin, *n* (%)								
0 mg/1000 kcal	Ref		Ref		Ref		Ref	
0–0.32 mg/1000 kcal	0.91 (0.77–1.08)	0.279	0.86 (0.71–1.03)	0.103	0.79 (0.63–0.99)	0.039	0.77 (0.61–0.97)	0.029
>0.32 mg/1000 kcal	0.83 (0.71–0.97)	0.017	0.76 (0.63–0.92)	0.006	0.73 (0.59–0.90)	0.004	0.69 (0.55–0.86)	0.002

Ref, reference; OR, odd ratio; CI, confidence interval; Ow, overweight; Ob, obesity; Central OB, central obesity.

[a] Adjusted for age, gender, race.

[b] Adjusted for age, gender, race, education, PIR, household education, tobacco exposure, birth weight, mother smoked when pregnant, and total cholesterol.

### Association between flavanones intake and ow/Ob and CO in children and adolescents stratified by age, and gender

To further analyze whether this association exists in children and adolescents with different status, subgroup analyses were performed (Table 3). Flavanones (level > 1.74) were associated with lower odds of Ow/Ob in children and adolescents aged 6–11 years in males (*p* = 0.082, *p* = 0.015, respectively). Eriodictyol (0<level ≤ 0.09 or/and level > 0.09) was associated with lower odds of Ow/Ob in children and adolescents aged 6–11 years in males (*p* = 0.034, *p* = 0.011, *p* = 0.003, respectively). Hesperetin (0<level ≤ 7.17 or/and level > 7.17) was associated with lower odds of Ow/Ob in children and adolescents aged 12–18 years in males or females (*p* = 0.029, *p* = 0.013, *p* = 0.030, respectively). Naringenin (level > 0.32) was associated with lower odds of Ow/Ob in children and adolescents aged 12 ~ 18 years in males (*p* = 0.100, *p* = 0.051, respectively). In addition, flavanones (level > 1.74) were associated with lower odds of CO in children and adolescents aged 12 ~ 18 years in males (*p* = 0.012, *p* = 0.004, respectively). Eriodictyol (0<level ≤ 0.09 or/and level > 0.09) was associated with lower odds of CO in children and adolescents aged 12–18 years in males (*p* = 0.048, *p* = 0.064, *p* = 0.061, respectively). Hesperetin (0<level ≤ 7.17 or/and level > 7.17) was associated with lower odds of CO in children and adolescents in males or females (*p* = 0.023, *p* = 0.079, respectively). Naringenin (0<level ≤ 0.32 or/and level > 0.32) was associated with lower odds of CO in children and adolescents aged 6–11 or 12 ~ 18 years in males (*p* = 0.076, *p* = 0.018, *p* = 0.006, respectively).

**Table 3:** Association between flavanones intake and Ow/Ob and Central OB in children and adolescents stratified by age, and gender.

Variables	Ow/Ob				Central OB			
	OR (95%CI)	*p*	OR (95%CI)	*p*	OR (95%CI)	*p*	OR (95%CI)	*p*
Subgroup I: age	6–11 years		12–18 years		6–11 years		12–18 years	
Flavanones, *n* (%)								
0 mg/1000 kcal	Ref		Ref		Ref		Ref	
0–1.74 mg/1000 kcal	0.91 (0.71–1.16)	0.443	0.92 (0.68–1.24)	0.578	0.74 (0.51–1.07)	0.105	0.86 (0.63–1.16)	0.306
>1.74 mg/1000 kcal	0.83 (0.68–1.02)	0.082	0.84 (0.67–1.07)	0.162	0.80 (0.59–1.08)	0.137	0.68 (0.51–0.92)	0.012
Eriodictyol, *n* (%)								
0 mg/1000 kcal	Ref		Ref		Ref		Ref	
0–0.09 mg/1000 kcal	0.75 (0.58–0.98)	0.034	0.73 (0.51–1.05)	0.091	0.81 (0.58–1.11)	0.186	0.96 (0.62–1.48)	0.834
>0.09 mg/1000 kcal	0.67 (0.49–0.91)	0.011	0.86 (0.64–1.14)	0.289	0.80 (0.51–1.26)	0.328	0.70 (0.50–0.99)	0.048
Hesperetin, *n* (%)								
0 mg/1000 kcal,	Ref		Ref		Ref		Ref	
0–7.17 mg/1000 kcal,	0.81 (0.63–1.04)	0.101	0.74 (0.56–0.97)	0.029	0.79 (0.55–1.14)	0.201	0.77 (0.52–1.13)	0.173
>7.17 mg/1000 kcal,	0.83 (0.63–1.08)	0.165	0.90 (0.68–1.18)	0.434	0.84 (0.60–1.18)	0.316	0.80 (0.60–1.06)	0.115
Naringenin, *n* (%)								
0 mg/1000 kcal	Ref		Ref		Ref		Ref	
0–0.32 mg/1000 kcal	0.89 (0.70–1.12)	0.309	0.96 (0.74–1.25)	0.766	0.73 (0.52–1.03)	0.076	0.87 (0.64–1.18)	0.356
>0.32 mg/1000 kcal	0.87 (0.69–1.08)	0.207	0.81 (0.63–1.04)	0.100	0.80 (0.60–1.08)	0.136	0.69 (0.50–0.93)	0.018
Subgroup II: gender	Male		Female		Male		Female	
Flavanones, *n* (%)								
0 mg/1000 kcal	Ref		Ref		Ref		Ref	
0–1.74 mg/1000 kcal	1.12 (0.92–1.36)	0.237	0.75 (0.55–1.02)	0.066	0.81 (0.60–1.10)	0.173	0.80 (0.58–1.11)	0.175
>1.74 mg/1000 kcal	0.80 (0.66–0.96)	0.015	0.85 (0.66–1.11)	0.227	0.71 (0.57–0.89)	0.004	0.76 (0.51–1.14)	0.183
Eriodictyol, *n* (%)								
0 mg/1000 kcal	Ref		Ref		Ref		Ref	
0–0.09 mg/1000 kcal	0.62 (0.46–0.84)	0.003	0.86 (0.60–1.23)	0.394	0.71 (0.50–1.02)	0.064	1.01 (0.67–1.54)	0.947
>0.09 mg/1000 kcal	0.76 (0.54–1.07)	0.117	0.79 (0.57–1.10)	0.164	0.71 (0.49–1.02)	0.061	0.79 (0.52–1.20)	0.268
Hesperetin, *n* (%)								
0 mg/1000 kcal	Ref		Ref		Ref		Ref	
0–7.17 mg/1000 kcal	0.86 (0.67–1.11)	0.235	0.70 (0.51–0.96)	0.030	0.92 (0.67–1.27)	0.614	0.68 (0.45–1.05)	0.079
>7.17 mg/1000 kcal	0.74 (0.58–0.94)	0.013	1.01 (0.77–1.33)	0.923	0.70 (0.52–0.95)	0.023	0.94 (0.60–1.47)	0.784
Naringenin, *n* (%)								
0 mg/1000 kcal	Ref		Ref		Ref		Ref	
0–0.32 mg/1000 kcal	1.09 (0.92–1.30)	0.327	0.77 (0.57–1.05)	0.094	0.85 (0.65–1.11)	0.219	0.76 (0.54–1.06)	0.108
>0.32 mg/1000 kcal	0.82 (0.67–1.00)	0.051	0.84 (0.65–1.10)	0.197	0.67 (0.51–0.89)	0.006	0.80 (0.55–1.18)	0.256

Ref, reference; OR, odd ratio; CI, confidence interval; Ow, overweight; Ob, obesity; Central OB, central obesity. Subgroup I: adjusted for gender, race, education, PIR, household education, tobacco exposure, birth weight, mother smoked when pregnant. Subgroup II: adjusted for age, race, education, PIR, household education, tobacco exposure, birth weight, mother smoked when pregnant.

## Discussion

This study aims to investigate the association of flavanones intake with Ow/Ob and CO in children and adolescents. We found that high intake of flavanones, eriodictyol, hesperetin, and naringenin were associated with lower odds of Ow/Ob in children and adolescents. In addition, high intake of flavanones, eriodictyol, and naringenin were associated with lower odds of CO in children and adolescents. Further subgroup analyses showed that among all the different subgroups, high intake of flavanones was associated with lower odds of Ow/Ob and CO in children and adolescents.

Phytochemicals including flavonoids, phenolic compounds, carotenoids, alkaloids, and organosulfur compounds (29, 30), have been reported to be associated with weight control (21, 31). For example, an analysis from a sample of Iranian school-age children elaborated that a higher load of phytochemicals in the diet was associated with a lower risk of Ow/Ob (32). In addition, habitual flavonoid intake has been reported to be inversely associated with Ow/Ob risk or changes in adiposity measures (21, 33). Flavonoids also could modulate adipokines, which were involved in obesity and inflammation (34, 35). Flavanones, also called citrus flavonoids, are an important series of flavonoids, mainly including hesperidin, naringenin, and eriodictyol (36). Studies in Korean adults have found that a high intake of flavanones may be associated with a decreased body fat percentage and CO in women (22). In addition, naringenin, eriodictyol, and hesperetin have emerged as promising therapeutic agents for the control of Ow/Ob and the treatment of metabolic disorders (24, 37). Lopez-Almada et al. (24) found that naringenin may be involved in inhibiting the onset and progression of Ob and its comorbidities through multiple pathways such as insulin resistance (IR), inflammation, OS, macrophage infiltration, dyslipidemia, and hepatic steatosis. Kwon et al. (38) found that dietary eriodictyol can prevent Ob and related metabolic diseases caused by diet, including hepatic steatosis, dyslipidemia, IR, and inflammation. Yoshida et al. demonstrated that citrus flavonoids hesperetin and naringenin could directly block TNF-alpha-stimulated FFA secretion (39). Phenolic compounds, among which isoflavones, may inhibit appetite through a modulation of the pattern of gene expression of peripheral and central peptides involved in feeding control, thus further supporting their importance as anti-obesity agents (40). Based on the above conclusions, in this study, we demonstrated that high intake of flavanones, eriodictyol, hesperetin, and naringenin were associated with lower odds of Ow/Ob in children and adolescents. In addition, high intake of flavanones, eriodictyol, and naringenin were associated with lower odds of CO in children and adolescents.

Several mechanisms have been proposed to describe the association between high intake of flavanones and the lower odds of Ow/Ob and CO in children and adolescents. Studies have shown that several transcription factors play important roles in activating lipogenesis, including peroxisome proliferator-activated receptor gamma(PPAR-γ) and CCAAT/enhancer binding protein(C/EBPs) (41). Citrus flavonoids extracts can inhibit fat accumulation and intracellular triglycerides, reducing the PPAR-γ expression (42). Tumor necrosis factor-α (TNF-α) stimulates free fatty acid (FFA) secretion through adipocyte lipolysis, and increased plasma levels of FFA promote insulin resistance (39, 43). The hesperetin and naringenin inhibit NF-κB and ERK pathways, which in turn suppress TNF-α-stimulated FFA secretion and thereby inhibiting adipocyte lipolysis (39).

This study is the first to demonstrate that a diet loaded with high flavanones was associated with a decreased risk of Ow/Ob and CO in children and adolescents. Flavanone is a citrus phytochemical with health-promoting properties associated with a lower risk of Ob. Given that the incidence rates of Ow and Ob in children and adolescents are continuing to increase globally, it is imperative that current public health strategies should include education about flavanones intake. Encourage children and adolescents to increase their intake of flavanones, such as fruits, vegetables, cereals, legumes, dark chocolate, coffee, tea, and wine, among others.

The present study has several limitations. First, because of the cross-sectional design of this study, it cannot identify a causal link between the exposure and the outcome. Second, this study may be limited by other uncollected confounders, such as genetic Ob. Third, the tobacco exposure, birth weight, and physical activity levels were collected using self-report; thus, recall bias is inevitable.

## Conclusion

A diet loaded with high flavanones was associated with lower odds of Ow/Ob and CO in children and adolescents. Further studies particularly the prospective ones are needed to confirm these findings.

## Data availability statement

Publicly available datasets were analyzed in this study. This data can be found at: NHANES database, https://wwwn.cdc.gov/nchs/nhanes/.

## Ethics statement

The requirement of ethical approval was waived by The Sixth Affiliated Hospital of Harbin Medical University for the studies involving humans because NHANES is a publicly available dataset and was approved by the NCHS Ethics Review Board. The studies were conducted in accordance with the local legislation and institutional requirements. Written informed consent for participation in this study was provided by the participants' legal guardians/next of kin.

## Author contributions

YL: Conceptualization, Project administration, Supervision, Writing – original draft, Writing – review & editing. ZL: Writing – review & editing, Methodology, Investigation, Formal analysis, Data curation. NW: Writing – review & editing, Project administration, Conceptualization.

## Footnotes

^1^ http://www.cdc.gov/nchs/nhanes.htm

^2^ https://www.cdc.gov/growthcharts/clinical_charts.htm
