# Supplementary material for: Association between intake of flavanones and the overweight/obesity and central obesity in children and adolescents: a cross-sectional study from the NHANES database
Source: Front Nutr. 2024 Jul 17;11:1430140. doi: 10.3389/fnut.2024.1430140 (PMC11288817; doi:10.3389/fnut.2024.1430140)
Supplement: Supplementary file 1 [file Table_1.DOCX]

Table S1 Comparison for missing data before and after data interpolation

| Variables | Before interpolation | After interpolation | Statistics | *P* |
| --- | --- | --- | --- | --- |
| Household education, n (%) |  |  | χ^2^=1.91 | 0.167 |
| <high school degree | 1566 (19.02) | 1639 (19.29) |  |  |
| ≥high school degree | 4169 (80.98) | 4331 (80.71) |  |  |
| Tobacco exposure, n (%) |  |  | χ^2^=0.19 | 0.664 |
| No | 5082 (87.17) | 5134 (87.14) |  |  |
| Yes | 825 (12.83) | 836 (12.86) |  |  |
| Education, n (%) |  |  | χ^2^=1.74 | 0.187 |
| <9th grade | 4837 (78.46) | 4842 (78.49) |  |  |
| ≥9th grade | 1128 (21.54) | 1128 (21.51) |  |  |

χ^2^: Chi-square test.

Table S2 Screening for potential covariates of Ow/Ob

| Variables | OR (95%CI) | *P* |
| --- | --- | --- |
| Age | 1.00 (0.99-1.02) | 0.817 |
| Gender |  |  |
| Male | Ref |  |
| Female | 0.92 (0.78-1.09) | 0.348 |
| Race |  |  |
| Non-Hispanic White | Ref |  |
| Non-Hispanic Black | 1.41 (1.17-1.71) | <0.001 |
| Others | 1.48 (1.24-1.78) | <0.001 |
| Education, n (%) |  |  |
| <9th grade | Ref |  |
| ≥9th grade | 0.80 (0.68-0.94) | 0.009 |
| Household education |  |  |
| <high school degree | Ref |  |
| ≥high school degree | 0.62 (0.49-0.78) | <0.001 |
| PIR |  |  |
| <1.0 | Ref |  |
| ≥1.0 | 0.64 (0.55-0.74) | <0.001 |
| Unknown | 0.93 (0.65-1.34) | 0.689 |
| Tobacco exposure |  |  |
| No | Ref |  |
| Yes | 1.34 (1.05-1.70) | 0.019 |
| Physical activity |  |  |
| No | Ref |  |
| Yes | 0.96 (0.73-1.27) | 0.760 |
| Unknown | 1.03 (0.82-1.30) | 0.799 |
| Birth weight |  |  |
| <5.5 | Ref |  |
| 5.5-8.9 | 1.13 (0.83-1.54) | 0.443 |
| ≥9 | 1.45 (1.01-2.09) | 0.045 |
| Unknown | 0.90 (0.66-1.23) | 0.504 |
| Mother smoked when pregnant |  |  |
| No | Ref |  |
| Yes | 1.38 (1.05-1.82) | 0.020 |
| Total energy | 1.00 (1.00-1.00) | 0.144 |
| Fiber | 0.99 (0.98-1.00) | 0.168 |
| Total fat | 1.00 (1.00-1.00) | 0.679 |

Ref: reference, OR: odd ratio, CI: confidence interval.

Ow: overweight; Ob: obesity; PIR: poverty income ratio.

Table S3 Screening for potential covariates of central OB

| Variables | OR (95%CI) | *P* |
| --- | --- | --- |
| Age | 0.98 (0.97-0.99) | **0.046** |
| Gender |  |  |
| Male | Ref |  |
| Female | 1.06 (0.91-1.23) | 0.427 |
| Race |  |  |
| Non-Hispanic White | Ref |  |
| Non-Hispanic Black | 1.27 (1.01-1.61) | **0.044** |
| Others | 1.42 (1.13-1.80) | **0.004** |
| Education, n (%) |  |  |
| <9th grade | Ref |  |
| ≥9th grade | 0.61 (0.50-0.73) | **<0.001** |
| Household education |  |  |
| <high school degree | Ref |  |
| ≥high school degree | 0.67 (0.53-0.86) | **0.003** |
| PIR |  |  |
| <1.0 | Ref |  |
| ≥1.0 | 0.63 (0.50-0.81) | **<0.001** |
| Unknown | 0.80 (0.52-1.22) | 0.287 |
| Tobacco exposure |  |  |
| No | Ref |  |
| Yes | 1.70 (1.27-2.27) | **<0.001** |
| Physical activity |  |  |
| No | Ref |  |
| Yes | 0.78 (0.56-1.08) | 0.130 |
| Unknown | 0.88 (0.64-1.22) | 0.444 |
| Birth weight |  |  |
| <5.5 | Ref |  |
| 5.5-8.9 | 0.88 (0.62-1.25) | 0.471 |
| ≥9 | 1.35 (0.83-2.19) | 0.221 |
| Unknown | 0.57 (0.39-0.85) | **0.006** |
| Mother smoked when pregnant |  |  |
| No | Ref |  |
| Yes | 1.65 (1.33-2.04) | **<0.001** |
| Total energy | 1.00 (1.00-1.00) | 0.175 |
| Fiber | 1.00 (0.98-1.01) | 0.705 |
| Total fat | 1.00 (1.00-1.00) | 0.442 |

Ref: reference, OR: odd ratio, CI: confidence interval.

CO: central obesity; PIR: poverty income ratio.
